# Remarks on the Condition of the System, between the Period of Overpowering Stimulation and Reaction

**Published:** 1858-11

**Authors:** Orrin Smith

**Affiliations:** Late Professor of Obstetrics and Diseases of Women and Children in the University of Vermont; Chicago, Ill.


THE CHICAGO

MEDICAL JOURNAL.

VOL. I. NOVEMBER, 1858. No. 11.

### ORIGINAL COMMUNICATIONS.

ARTICLE I.

### REMARKS ON THE CONDITION OF THE SYSTEM, BETWEEN THE PERIOD OF OVERPOWERING STIMULATION AND REACTION.

BY ORRIN SMITH, M. D., LATE PROFESSOR OF OBSTETRICS AND DISEASES OF WOMEN AND CHILDREN IN THE UNIVERSITY OF VERMONT.

(Read before the Chicago Medical Society.)

MR. PRESIDENT AND GENTLEMEN OF THIS SOCIETY,—

Permit me to call your attention to that interval of time between the stage of excitement and the stage of reaction.

We all acquiesce in the proposition that the blood answers certain intentions in the animal existence; among which are;

1*st.* To provide material for the nutrition and maintenance of the several parts of the body.

2*d.* To convey oxygen to the several parts, to enable them to perform their functions, and combine with effete or refuse material.

3*d.* To bring from the several parts those refuse matters, and convey them to where they may be discharged from the body.

And it is also universally agreed that the blood is propelled to all parts of the body by the *agency* of nervous power operating upon the striped and unstriped muscular tissue. So that after we have canvassed all causes of vital action, examined the functions of digestion, nutrition and assimilation, and reproduction of tissues, we necessarily fall back upon that something which all call vital force or nervous power, and locate it in the brain and nervous centres, as furnishing the agency by which these phenomena are manifested. And whether this vital power, for the performance of these and other functions, is, or resembles galvanism, electricity, or electro-magnetism, or any form of philosophic power, it is not my present purpose to inquire; or when it will be demonstrated in what nervous power or agency consists. It is sufficient for our present purpose to inquire, how is this vital power, which gives action to every organ, supplies the power for the performance of every function, produced? Or rather, what are the favorable and unfavorable circumstances for the development and perpetuation of this power, so far as they are known? For it may be borne in mind that we cannot make any very lucid claims to understand this subject, and demonstrate it as a science. Still many of the phenomena of nervous power are understood and appreciated by the profession. For instance, when, in tracing embryotic dovelopment, we say that the nervous structure is early and largely developed, and that through the periods of fœtal and infantile existence this development is proportionally larger than in adult years, and when we assert that during these periods the nervous structure is more sensitive to impressions, both physiological and pathological, we only assert a truth which is well known to the profession, and one which is probable no future discovery will repudiate.

Neither should we need to be reminded that this exalted estate of the nervous sensibility can only be maintained, except by a proportionally larger supply of the blood being sent to the nervous centres. It is also conceded that the operations of the mind, mental effort, are but functions of the brain and nervous structure or tissue. Hence, mental energy and emotion, either of childhood or adult years, implies that not only the nervous power is in an exalted state, but the vascular organs supplying the nervous structure are also in a state of excited action or excitement, proportioned to the amount of demand for that nervous power. And this exalted state of the nervous and vascular structure is to be continued until the object of the demand is attained, or the system becomes exhausted by its efforts, and relaxes into a state of inaction or repose, proportioned in length to the amount and continuance of the previous excitement, and the ability of the system to recuperate itself by restoring the lost energy of the nervous system and rebalancing the circulation. And although in philosophy it is laid down as an axiom, that action and reaction are equal, and in physiology that subsequent excitement is usually proportioned to the previous depression, neither of these propositions explain the operations of, or cover, the period to which our attention is now directed. But it is to that intervening period before alluded to, where *inanition*, proportioned to the previous excitement or demand for vital force, *takes* the place of excitement and *normal action* also, to which I wish more especially to refer, for the purpose of deciding as to its practical bearings in the everyday duties of our profession.

In our profession it is well understood, that in active inflammation, depletion and antiphlogistics proportioned to the demand are requisite; in congestion, a restoration of the normal condition of the circulatory function is the indication; and our rules for treating these cases are well defined, and acquiesced in by the profession generally. And when errors occur in treating such cases, they are usually the result of mistaking the intensity or severity of the pathological condition, or the potency of the remedies indicated, and not the indications to be answered or means to be used. But this, I apprehend, is not always the case in that period of pathological action, if it can be so called, which we have under consideration—a period of time of uncertain length, which is measured by the circumstances of the *cause*, the restorative power of the system, and the means employed for the restoration of the normal condition; and according as these may affect the case, it may be of trifling account, or important as life, with all intermediate conditions and complications. In many cases to which I allude, the symptoms may be so prominent, as to make the condition almost self-evident; while, in others, it may be quite different by our ordinary methods of reasoning. For instance, we see this condition portrayed when some sudden intelligence or fright operates as an overpowering stimulus, and blanches the cheek of the sensitive female, and she, pale, lifeless and senseless, falls to the ground, an inanimate mass of yet living sensibilities, and all in abeyance to nature's physical laws. It is true that here the whole range of the exciting cause may have been as rapid as thought, or of duration scarcely longer than the lightning's flash; or, in other cases, the effect of that disturbed state of the electricity of the atmosphere may annihilate the nervous power of the system, by what is called the lightning's stroke; and in these cases, where the concussion has produced none but functional lesions of organs, the condition of the system is much the same as in the former supposition, only more rapid, perhaps more violently produced, and may, or may not, be more serious in its consequences.

Now what is here the pathological condition of patients in the two cases? Why, simply this, the overpowering stimulus of thought in the one case, and the stimulus of the electric fluid in the other, have suspended the functions of the brain, the nervous power, and, as a consequence, the muscular tissue of both voluntary and involuntary motion is suspended, and temporarily or permanently, according to the severity of the exciting cause, treatment, and other conditions before named.

Here we find the blood no longer pressed upon the brain with sufficient force to enable the brain to keep up its function of furnishing the nervous power, and, consequently, the muscular tissues of the heart and arteries no longer presses the blood upon the brain with sufficient force, and, as a consequence, the muscles of voluntary motion have entirely lost their contractility, and the body being left to action of physical laws, assumes a horizontal position.

What next do we see? We see also the blood leave the surface, receding from the capillaries, and falling back into the larger vessels, and why? Simply to make, by the force of gravity, that pressure upon the brain which is lost by the want of muscular contraction. And this resumption of the functions of the brain may be induced so long as the endangium supplies the necessary vitality of the blood to prevent coagulation in the heart and larger arteries; and when by the force of gravity the blood exercises a sufficient amount of pressure upon the brain, it proceeds to give off that vital energy which is the source of contraction, and consequently the functions of respiration and circulation are resumed. It is true that in the restoration of these cases we can assist by sprinkling the face and chest with cold water, by quick, smart blows upon the muscular and flat surfaces of the body, to rouse the dormant nervous power of the system. These last are simply stimulating to whatever there may be of nervous power in the system; while the recession of blood from the capillaries and consequent pressure of blood upon the brain *engenders* or *produces* vital action, and is *nature's* own remedy.

We have the same condition of abated nervous energy or power when the system is brought under the full therapeutic effect of tobacco, antimony, veratrum viride, colchicum, etc., etc., modified by the peculiar specific properties of the agents named, except the property of mere depressors of nervous power.

Again, a man receives a severe injury, extensive laceration, bruising of soft parts, or fractures of bones, then follows the stage of depression known as "*the shock*," which is a constitutional effect immediately following the injury; and our best modern surgeons and pathologists say that it consists in "a disturbance of the circulatory, respiratory and nervous systems," "the harmony of the great organs becomes deranged;" which is all very true; but in this description of phenomena is the true pathological condition indicated? In my opinion it is not, and therefore the true principles of treatment are not shadowed forth in the pathological description. The shock, to my mind, is simply the abated or lessened power or physical function of the brain, and the disturbed respiration and circulation are only, a *consequence*, a want of motive power derived *from* the brain. Let us look a little farther and see what follows, and to prevent any peculiar notions of my own from warping my judgment in description, let me quote from Erickson's Surgery, one of our best and most popular modern works. He says: "On the receipt of the injury the sufferer becomes cold, faint, and trembling; the pulse is small and fluttering; there is mental disquietude and depression; the disturbed state of the mind revealing itself in the countenance, and in the incoherence of speech and thought; the surface becomes covered with a cold sweat; there is nausea; perhaps vomiting, and a relaxed state of the sphincters. In extreme cases, the depression of power is so great as to terminate in death."

What does all of this formidable train of symptoms indicate? To me, a want of nervous power sufficient to keep up the tonicity of the system; the necessary pressure of blood upon the brain.

How much does this differ from the pathological condition before named, except in degree and modifications of pain and mental excitement, perhaps? When carefully considered, I will say, not essentially, and the differences are only the superadded conditions to the great pathologic cause, the suspended function of ennervation. And these phenomena are dependent, not upon concussion, but the effect of the stimulation in suspending the functions of the brain and large nervous centres. Or let an artery be wounded, and the blood flow until the brain fails to receive the necessary pressure, and what follows? Why, simply the same symptoms, with some slight variation, but not enough to make us doubt our diagnosis in the preceding cases, but rather to confirm it. We find here the same general phenomena as in ennervation following mental emotion, and nature left to herself, pursues the same restorative process. It is true, in the last named supposition, before complete restoration, the digestive function may be called upon to supply the deficiency of the vital fluid; but we must have reaction previous to this, and therefore it does not come within the limits of our investigation.

We also find the same condition of the system when we find our patient sunken, prostrate from menorrhagia, or uterine flooding from any cause. And this remark is as applicable to floodings in parturition, either premature or at term, as to any condition which could be named. And permit me to add, that when this principle of causing blood to press with sufficient force upon the brain shall be properly understood and practised by the profession generally, we shall have disarmed the lying-in room of one-half of its terrors, as they at present occur.

Death from uterine flooding will be of very rare occurence! Retained placentas seldom heard of! And hour-glass contractions almost a myth. The occurrence of uterine inertia lessened. And the time of parturition materially shortened! as it has been during the last twenty-five years, by a general substitution of the recumbent, or rather semi-recumbent, position for the sitting posture during labor. We will also find the tampon, as an appendage to the lying-in room, *seldom* required, and the transfusion of blood *uncalled* for!

I am aware that there is a tone or spirit in these remarks which should be exercised with caution, that one is liable to be led headlong by his own enthusiasm; but I trust I have sufficiently investigated this subject so as to speak only of legitimate deductions from properly investigated causes; and if so, I shall be pardoned by all lovers of medical science for any apparent over-zealous remarks.

In parturition, the physical exertion long continued exhausts the nervous power which supplies the muscular tissue, and it is quite often the case that labor is suspended, while the patient sleeps and rests, to restore the lost vitality; and when, in approaching this stage, the pains become shorter, and the intervals longer, the phenomena only indicate that the brain and nervous centres are not receiving that amount of pressure which is necessary for the performance of the functions of animal life and parturition added.

Long continued muscular exertion from any cause produces the same physiological result. The question may be properly asked, whether it is not the exhaustion of the muscular tissue itself, instead of the nervous power, which gives the want of contractile force to the muscles. This and many other collateral questions are too long to discuss at length in this paper; still I will say that tonic spasm, for weeks or months even, does not abate itself by contraction, nor is it abated so long as the nervous pathological condition which causes it is in existence. And that clonic spasms, when they, by the force contractions and relaxations, have depleted sufficiently the *nervous* irritability, always end in repose. Again, the knowledge of this principle should be with us in the treatment of acute disease. For we may exhaust the nervous or vital power of the system, while local or general acute disease is unsubdued; and the same may be said of some febrile diseases.

Let me take another instance for illustration, and I will take a case whose pathology does not appear to be closely defined in our text books, or whose pathology is not generally understood alike by the profession, and it is one which will be easily appreciated if my present position is correct; I allude to *coup de soleil* or sun-stroke. Here we find this same physiological condition of the great nervous centres, and why? It is for the reason that the heat has suddenly relaxed the capillaries, and perhaps, indeed, muscular exertion is added, to exhaust nervous power; while the relaxed capillaries, by suddenly admitting blood from the larger vessels, drain the blood from the brain, so that it fails to perform its function. True *coup de soleil*, I apprehend, is always distinguishable from, and should never be confounded with, apoplexy, or congestion of the brain. It is a grievous error to mistake one for the other; yet I infer that it is quite common to regard *sun-stroke* as a *congestion* of the brain: a worse error could not be made, as it totally misdirects the treatment, if my pathological opinions are correct. Hence, I will say, let apoplexy, congestion of the brain, and *coup de soleil*, be treated according to their several pathological indications.

When the physician steps between the patient and the recuperative operations of nature, and endeavors to set up a new process which *he* may term restorative, there can be no matter of *doubt* about the value of his prescriptions. This may be best illustrated by a case which occurred in our streets only a few days since, of an Irish laborer, who was the subject of *sun-stroke* while employed in grading one of our public streets. The treatment employed by "Biddy," his wife, may be worth detailing, as illustrating the fertility of genius in prescription; and certainly it was not so far-fetched as some *regular* prescriptions have been. When news was brought to "Biddy" that her husband was sun-struck, she hurried to pour and carry to him some chicken broth which she was making for his dinner, and gave the poor man some "sups" of the same. When she bethought her, that her good man always liked a smoke after eating; so she propped him in a sitting posture against the fence and hasted to bring his pipe, but her absence was to long for the drained brain of Pat, and she returned to find him dead—a martyr, perhaps, to her unskilful prescription; but neither herself nor the bystanders saw any impropriety in draining an exhausted brain of its remaining vitality, and poor Pat was buried in due time, because nature's chosen agent, position, for his recovery, was denied him, although in kindness.

But we must expect unscientific treatment in *coup de soleil* until its true pathology is understood and impressed upon the public mind; and when this is the case, we shall be content to let the body lie supine, with head pendant, and let the blood *leave* the surface as nature orders, without attempting by rubefacients and external stimulants to *keep* the blood upon the surface and away from the deep-seated vessels which make direct communication with the brain. And we shall understand that the most approved and popular remedies of our text books, as ammonia, alcohol, by the mouth or rectum, when the patient cannot swallow, can only operate to stimulate that nervous energy by indirect means which can be accomplished directly by *position*. These remedies are all well enough, but a *direct* nervous power derived from due pressure of blood upon the brain, is worth more than *all* other remedies in these cases. And nature emphatically points the way to lay the head low, give free circulation of air, *let* the blood *leave* the surface until reaction takes place; these are the first steps to reaction, and with them many a sun-struck man will, in due time, resume his avocation, when, with other treatment, he has done his last work.

And where but in this same interval of time, between the period of excitement and reaction, shall we look for the cause of death in those persons who, in times of severe epidemics, die of fear and its consequences, as has been observed in cholera, plague, yellow fever; and where shall we find the pathological indication of cure but in the course I have named, and to remove, if possible, the mental emotion which is destroying the nerve power.

Again, the army surgeon has a wide field for studying this ennervation of which I am speaking; and, perhaps, at no moment during the battle does so many combatants die as at the moment that the "shout of victory rends the air," when each prostrate hero raises his head to see "*who flies*," when the blood fails to make the necessary pressure upon the brain, and he falls back a corpse, and from wounds not necessary fatal, but because they have drained him of his *blood*, and its consequences follow.

I will illustrate the practical bearings of *position* upon the period of time of which I am speaking, by giving the details, in part, of a single case which occurred in my practice; one from the class of every-day occurrences, important, because fully demonstrating the grounds of my assumption.

Case 1. In February, 1854, I was called to see a Mrs. S., at nine o'clock a.m.; found she had a miscarriage at four p.m., the day previous, and that she had flowed profusely through the night, so that, in the judgment of her husband and two ladies of the neighborhood, she had fainted more than fifty times during the interval. My patient being at a distance of some seven miles, I was prepared to find a desperate case on arrival; and was not dissapointed, when I was told by the nurses that she had been dead for half an hour. They were the only persons present, her husband having come for me, and did not keep up with me in my rapid drive to his house. And they further said, they were waiting for him to come and say how he would have her "*laid out*." On my expressing a doubt of her having been dead half an hour, they affirmed that it *must be* twenty minutes, and they thought half an hour, since she had breathed or shown any signs of life. I repaired immediately to the room, where the body lay upon the bed, with her arms folded across her chest and her eyelids kindly drawn down until they nearly covered the eyeball, and surely, so far as any physical signs could disclose, on a hasty examination, she was dead. My hand passed over the region of the heart, discovered no pulsations; she had bled, in a horizontal position, until all evidence of vitality had ceased—I had full reason to think from twenty minutes to half an hour; for her attendants were perfectly calm and cool, and were persons of strict integrity. Under this forbidding aspect, I immediately decided to put in practice a principle of causing blood to *press on the brain by gravity* instead of vital power, which seemed most thoroughly absent. I immediately removed the pillow which was under her head, which let the posterior part of her head fall over the edge of her feather bed and rest upon her mattress. I then raised the foot of the bed by putting a common chair under each foot-post—which must have raised her feet above the head some eighteen or twenty inches. I then directed each female attendant to rub a lower limb, in a manner to drive the blood from the extremity toward the brain, and to rub briskly; which, after *twice* telling me that *she was dead*, and asking me if *I was crazy*, I succeeded in getting them to do, while I performed the same manipulation upon the upper extremities, alternately. After pursuing this course some two or three minutes, perhaps (for we kept no record of time, only from memory), she gasped convulsively, like one of those deep, long-drawn gasps which we see as the last gasps of life. We briskly continued the rubbing, when, in less than a minute, she gasped again; and again, and again, at shorter intervals, until probably within five or six minutes, regular, free, quiet breathing was restored. When her breathing was well established, we desisted from the frictions. After breathing quietly some ten minutes, perhaps, she opened her eyes and recognized me with a faint smile. And I asked her if she wished her lips wet, and she faintly answered, yes, and I gave her a tea-spoonful or two of cold water, in which was a small amount of tincture of camphor, I not designing to get any medicinal effect from anything but position, in order to test fully, in an extreme case, my chosen remedy, *position*.

We now kept still, and she rested in a quiet slumber—perhaps half of an hour—when she awakened, and said she had been asleep; inquired how long I had been there, and if she was worse.

In the meantime, slight color had returned to her lips, and she had pulsations of the radial arteries. As I had abundant reason thus far to be satisfied with my remedial agent, I resolved to further test its power, and rely upon position alone in the end. Then I proceeded to turn out the clots from the vagina, when the uterus gave very good tonic contraction, making its tumor manifest above the pubes. I staid with my patient about four hours, during which time her soiled clothing was removed, and her bed made as comfortable as the pendant position of the head would allow. The uterus, in the meantime, kept up good tonic contraction, and the flooding had entirely ceased from her first assuming position, and a cloth wrung from cold water was kept cool and applied to the vulva, but was scarcely soiled by the discharge, so perfect was the contraction. Long before I left, reaction had taken place; she had learned the whole story of her illness, and expressed her gratitude for attentions. Without treatment, I think she would have had no further changes but the "*rigor mortis*," and the common changes of decomposition; and I left her, feeling that her life was as safe as patients generally, after ordinary confinement. I gave permission to lower the foot of the bed one half of its elevation that night, and the other half by successive depressions the succeeding twenty-four hours; with full directions for dietetic restoration of lost blood. I did not see her again for three weeks, when she called upon me at my place; and I then prescribed some bitter laxative and a preparation of iron, to complete the process of sanguification. She was speedily and completely restored.

I offer this single case as one covering the grounds of my remarks. It is indeed the most prominent one which has fallen under my observation; although I could quote many of less striking importance, based upon the same principles. And here let me digress to say, that I think it is a common custom in uterine floodings, with many, if not a majority of practitioners, to order their patient to lie still in bed, while blood and discomfort accumulates around them. After my previous remarks, you are prepared to hear me say, that all such cases can be moved, and even benefited, by lessening the danger by removal to easy positions and fresh beds as needed (and no precaution is needed in such cases, except to keep the head lower than the body); and often this very removal changes the aspect of the case—makes the patient comfortable, and removes the danger by checking the flooding.

I have seen cases of menorrhagia of the passive form, which, in the judgment of good practitioners, would terminate fatally in twenty-four hours, change their aspect entirely by depressing the head, or rather elevating the feet and body without change of medicine, and rapidly progress to recovery, the discharge lessening from the first hour of *position.*

Since I have known something of the value of position, I prize the general stimulants as of less value than formerly, in my practice; not that I would discard their use, but that I have less frequently needed them. Opiates, and many of the remedies used in floodings and hemorrhages, operate by giving a stimulated nervous energy, which is only a substitute for the inherent but depressed power of the system. The administration of vascular stimulants serve to press temporally the blood towards the vascular extremities, and in this peripheral determination the capillaries of the brain share largely, because not compressed by atmospheric pressure. This result is obtained by the stimulus causing the muscular tissue of the heart and arteries to contract upon the blood. Nervous stimulants, as camphor and ammonia, operate to rouse temporally the force of nervous energy, and in many cases either the vascular or nervous stimulants may be sufficient to press the blood upon the brain, so that it will give back its normal function. We sprinkle the face, the chest, the extremities with cold water, in cases of fainting and suddenly depressed vitality, to rouse by *reflex action* the normal condition.

We cool the surface to drive the blood to the deep-seated vessels, because they are in direct communication with, and press upon, the brain. And here, perhaps, is a formidable error in medical practice frequently; the question should be solved by the practitioner, whether the cool surface is not an effort of nature to restore vitality, or is it really a pathological condition to be counteracted by the physician? No one can hesitate a moment as to the importance of making the diagnosis, so long as it would dictate opposite modes and modifications of indication.

And again, let me say that we shall never have occasion to bring to our aid *reflex*, when we can employ *direct* power to produce the desired result—which I trust I have sufficiently shown—is always within our reach, and will accomplish more than any reflex action can accomplish; ready for any emergency; and is just so far preferable as any natural condition is preferable to an artificial one, however speciously devised.

In these brief remarks, I have aimed to speak only of general principles as they occur in practice, and tend to illustrate the *one point.* In many cases, a disjointed remark is all I have said upon a point, which, for full illustration, would have taken pages, well knowing that your professional knowledge would supply such apparent deficiency.

I may be asked why a certain amount of sanguinous pressure is *necessary* to enable the brain and nervous centres to perform their functions; I answer, it is sufficient if we note the fact *to be so.* Neither can we tell why healthy granulations only grow when covered with pus; or why pond lilies only grow when their roots lie deeply under the surface of the water; or why water itself congeals at 32°, or boils at 212°. One answer is sufficient for all; they are so, because they were made to be so.

CHICAGO, ILL., August 6th, 1858.

